# Pathogenicity and Antimicrobial‐Resistance Profiles of *Escherichia coli* Isolated From Faeces of Tibetan Pigs Reared Under Intensive Conditions

**DOI:** 10.1002/vms3.70899

**Published:** 2026-04-03

**Authors:** Runbo Luo, Lihong Zhang, Kexin Li, Yanan Zhong, Peng Shang, Suolang Sizhu, Md. F. Kulyar, Ruibing Cao

**Affiliations:** ^1^ College of Veterinary Medicine Nanjing Agricultural University Nanjing People's Republic of China; ^2^ College of Animal Science Xizang Agricultural and Animal Husbandry University Linzhi People's Republic of China; ^3^ Key Laboratory for Prevention and Control of Hydatid Disease in Xizang (Co‐constructed by Ministry and Province) Ministry of Agriculture and Rural Affairs Linzhi People's Republic of China; ^4^ College of Veterinary Medicine Gansu Agricultural University Lanzhou Gansu People's Republic of China; ^5^ Department of Regenerative Medicine State Research Institute Centre for Innovative Medicine Vilnius Lithuania

**Keywords:** antimicrobial phenotypic, *E. coli*, tibetan pig, virulence factors

## Abstract

**Background:**

Intensive indoor pig production systems can foster high antimicrobial exposure and dense animal contact, potentially enriching multidrug‐resistant (MDR) *Escherichia coli* (*E. coli*) that may enter wider One Health microbial networks.

**Objectives:**

This study investigated grower‐finisher Tibetan pigs managed under commercial conditions harbouring phylogenetically diverse *E. coli* lineages carrying extensive resistance and virulence determinants.

**Methods:**

Between 2021 and 2023, 280 rectal swabs were collected from nine mechanically ventilated farms; the isolated *E.coli* strains were subjected to phylogrouping, as well as detection of 13 virulence genes and 45 resistance genes, using multiplex PCR. Antimicrobial susceptibility of the isolates to 22 antibiotics was determined by the disk diffusion method.

**Results:**

Enteroaggregative *E. coli* accounted for 27.86% of isolates, and putative STEC/EHEC accounted for 7.14%, with *astA* being the most prevalent (27.86%). Phenotypically, 94.29% were resistant to amoxicillin and 68.57% to sulfonamides, while 82.86% satisfied the MDR definition; genotypically, *tetA* (70.71%) and *floR* (57.14%) dominated the resistance repertoire. Phylogroups A and B1 predominated, but resistance and virulence profiles were dispersed across all lineages, indicating horizontal gene flow rather than clonal expansion.

**Conclusion:**

Tibetan pigs represent a substantial reservoir of MDR *E. coli*, strengthening the rationale for farm‐level antimicrobial stewardship and integrated surveillance that links animal, environmental and human compartments. The study is limited by its cross‐sectional design, focus on a single province and animal host and the absence of parallel human or environmental isolates, which prevent inference of causal zoonotic pathways and should be addressed in longitudinal One Health investigations.

## Graphical abstract

Tibetan pig farms host diverse *Escherichia coli* strains that resist multiple antibiotics and carry important disease genes, such as *astA*. These traits jump easily between bacterial groups, marking intensive pig production as a key reservoir and signalling the need for integrated animal–human surveillance and more careful antibiotic use.

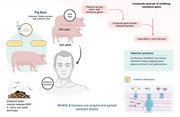

## 1 Introduction

Antimicrobial resistance in livestock‐associated bacteria is a recognized One Health concern because resistance determinants can circulate between animals, humans and the environment (Velazquez‐Meza et al. 2022). Pathogenic *Escherichia coli* (*E. coli*) is a primary zoonotic agent that can move from animals to humans through contaminated food or water. Consequently, it contributes substantially to the global burden of diarrhoeal disease (Foster‐Nyarko and Pallen 2022). To gain deeper insights into the genetic diversity among *E. coli* isolates, researchers have identified eight phylogenetic groups (A, B1, B2, C, D, E, F and cryptic clade I) (Bok et al. 2020), each characterized by unique genetic traits reflecting their evolutionary history and infective potential. The pathogenesis of *E. coli* is a complex interplay involving various virulence factors and their regulatory mechanisms (Dixit et al. 2004; Croxen and Finlay 2009), as well as interactions with other bacteria, which determine the ability of *E. coli* to cause infection.

Pigs are well‐known to port various pathogenic and non‐pathogenic *E. coli* strains in their faeces. The coexistence of multiple strains within pig herds creates favourable conditions for the rapid dissemination of *E. coli*, posing a significant challenge for animal health management (Bok et al. 2020). If these bacteria become prevalent in livestock, outbreaks may occur, leading to potential financial losses for farmers. Furthermore, studies have demonstrated that pathogenic *E. coli* strains in pig intestines can infect humans (Dohmen et al. 2023). This underlines the importance of controlling pathogen dissemination among animals, understanding their potential for cross‐species transmission and taking measures to prevent their transfer to humans.

The Tibetan pig is a unique breed adapted to the high‐altitude Qinghai Xizang Plateau. Its thick coat and robust build enable survival in extreme cold, and it provides an important protein source for local communities. Nevertheless, inadequate shelter, poor ventilation and crowding can foster unsanitary conditions that favour *E. coli* transmission. The abuse of antibiotics in livestock farming has given rise to antibiotic‐resistant bacteria, posing a significant threat to human health. This highlights the urgent need for efficient strategies to prevent and control bacterial infections in humans and animals. *E. coli* is prevalent in the pig farming industry and poses significant risk to human and animal health through foodborne illnesses. This study aimed to investigate the pathogenicity, antibiotic resistance characteristics and phylogenetic analysis of *E. coli* in Tibetan pig herds and to assess the associated public health risks. These data will inform the development of targeted prevention and control measures to mitigate *E. coli* infections in Tibetan pig herds.

## 2 Methods

### 2.1 Sample Collection, Isolation and Identification of *E. coli*

Between 2021 and 2023, 280 fresh faecal samples were collected from Tibetan pigs raised on nine farms in Xizang, China. These holdings were located in Linzhi (*n* = 3), Changdu (*n* = 3) and Shannan (*n* = 3) prefectures, 200–450 km apart, ensuring geographic representation across the central and south‐eastern Xizang area. The sample size was determined a priori using the prevalence study formula *n* = *Z^2^ P* (1 – *P*)/*d^2^*. Assuming a conservative multidrug‐resistant (MDR) *E. coli* prevalence of 50%, a 95% confidence level (*Z* = 1.96), and a margin of error being 0.06, the minimum sample size was 267, increased to 280 to account for potential culture attrition. This matches the sampling intensity used in recent porcine AMR investigations (Toya et al. 2025). The study farms were medium‐scale operations (300–500 pigs). Sampling targeted pre‐weaning nursery piglets aged 0–6 weeks (0.7–5 kg). Piglets were kept indoors in mechanically ventilated pens (6–7 piglets/pen; ≈0.5 m^2^/piglet) and fed a maize–soybean diet formulated to NRC recommendations. Rectal swabs were placed in Amies transport medium on ice and cultured within 4 h. Later, the samples were cultured on MacConkey agar plates (Haibo, China) and incubated overnight at 37°C under aerobic conditions after collection from the field. A single red colony was chosen from each sample for further purification and then subjected to conventional physiological and biochemical methods to identify it by incubation at 37°C for 24 h with specific reagents, with results interpreted based on colour changes. Positively identified *E. coli* strains were stored at −80°C in a broth containing 30% glycerol and Luria–Bertani (LB) broth.

### 2.2 Bacterial DNA Extraction

*E. coli* colonies were inoculated into nutrient broth media and grown overnight at 37°C, with agitation at 220 rpm for adequate aerobic growth. The boiling method was employed for DNA extraction due to its simplicity and effectiveness. This involved isolating and suspending a single colony in 300 µL of sterile water. The suspension was then heated to 98°C for 15 min in a heating block (Eppendorf, Germany). The suspension was centrifuged at 13,000 rpm for 2 min to separate the cellular debris from the DNA‐containing supernatant. The resultant supernatant, rich in bacterial DNA, was stored at −20°C for subsequent use in downstream applications.

### 2.3 Phylogenetic Analysis and Polymerase Chain Reaction (PCR) Detection of Virulence Genes

In order to study the phylogenetic diversity of *E. coli* strains isolated from Tibetan pigs, a multiplex PCR was employed to screen virulence genes and gauge zoonotic potential. Following previously described methods (Clermont et al. 2012), PCR amplification was conducted on each isolate to identify its phylogroup (A, B1, B2, C, D, E, F, cryptic clade I and unknown). In parallel, the PCR analysis was extended to detect a panel of virulence genes, including *escV*, *eae*, *bfpB*, *stx1*, *stx2*, *lt*, *stp*, *sth*, *invE*, *ipaH*, *aggR*, *pic* and *astA* in *E. coli* isolated from Tibetan pigs (Table 1). The PCR reactions were performed in a 25 µL reaction mixture containing 12.5 µL of 2× mix, 8.5 µL of ddH_2_O, 1 µL each of forward and reverse primers and 2 µL of DNA template. Electrophoresis was performed on 1.5% agarose gels with GreenViewer stain to visualize the PCR products (BIO‐RAD, USA). The gels were then imaged using the GelDoc1000 (Vilber Lourmat, France) for detailed analysis. The determination of diarrhoeagenic *E. coli* shall be made in accordance with Bonkoungou et al. (2012).

**TABLE 1:** Primers used to detect *E. coli* virulence genes. bp, base pairs; F, forward primer; R, reverse primer.

Gene	Primer	Sequence (5′ → 3′)	Amplicon size (bp)	GenBank
*escV*	F	ATT CTG GCT CTC TTC TTC TTA TGG CTG	544	FM180568.1
	R	CGT CCC CTT TTA CAA ACT TCA TCG C		
*eae*	F	ATT ACC ATC CAC ACA GAC GGT	397	Z11541.1
	R	ACA GCG TGG TTG GAT CAA CCT		
*bfpB*	F	GAC ACC TCA TTG CTG AAG TCG	910	FM180569.1
	R	CCA GAA CAC CTC CGT TAT GC		
*stx1*	F	CGA TGT TAC GGT TTG TTA CTG TGA CAG C	244	AE005174.2
	R	AAT GCC ACG CTT CCC AGA ATT G		
*stx2*	F	GTT TTG ACC ATC TTC GTC TGA TTA TTG AG	324	AE005174.2
	R	AGC GTA AGG CTT CTG CTG TGA C		
*lt*	F	GAA CAG GAG GTT TCT GCG TTA GGT G	655	CP000795.1
	R	CTT TCA ATG GCT TTT TTT TGG GAG TC		
*stp*	F	CCT CTT TTA GYC AGA CAR CTG AAT CAS TTG	157	AY342057.1
	R	CAG GCA GGA TTA CAA CAA AGT TCA CAG		
*sth*	F	TGT CTT TTT CAC CTT TCG CTC	171	CP000795.1
	R	CGG TAC AAG CAG GAT TAC AAC		
*invE*	F	CGA TAG ATG GCG AGA AAT TAT ATC CCG	766	AF283289.1
	R	CGA TCA AGA ATC CCT AAC AGA AGA ATC AC		
*ipaH*	F	TTG ACC GCC TTT CCG ATA CC	647	CP001064.1
	R	ATC CGC ATC ACC GCT CAG AC		
*aggR*	F	ACG CAG AGT TGC CTG ATA AAG	400	Z18751.1
	R	AAT ACA GAA TCG TCA GCA TCA GC		
*pic*	F	AGC CGT TTC CGC AGA AGC C	1111	AF097644.1
	R	AAA TGT CAG TGA ACC GAC GAT TGG		
*astA*	F	TGC CAT CAA CAC AGT ATA TCC G	102	AF161001.1
	R	ACG GCT TTG TAG TCC TTC CAT		

### 2.4 Antimicrobial Susceptibility and Antibiotic Resistance Genes Tests

The Kirby–Bauer (K–B) disk diffusion method for antimicrobial susceptibility testing adhered to the standards and interpretation criteria outlined by the Clinical and Laboratory Standards Institute. The methodology was employed to assess the susceptibility of 280 *E.coli* isolates against a panel of 22 antibiotics, including β‐lactams (ampicillin, amoxicillin, amoxicillin‐clavulanate, cephalothin, cefuroxime, ceftriaxone, cefepime, meropenem), aminoglycosides (gentamicin, streptomycin), tetracyclines (tetracycline, doxycycline), macrolides (erythromycin), quinolones (norfloxacin, ciprofloxacin), lipopeptides (polymyxin B), sulfonamides (sulfonamides, trimethoprim), nitrofurans (furazolidone, nitrofurantoin) and phenicols (chloramphenicol, florfenicol). *E.coli* ATCC 25922 served as the quality control strain in the analysis. An isolate was classified as MDR when it showed resistance to three or more antimicrobial classes. In the summary graphics, the exact breadth is represented as 3R, 4R, 5R and so forth, where 3R indicates resistance to exactly three classes, 4R to 4 classes and so on.

PCR amplification was used to detect antibiotic resistance‐coding genes according to the previously reported methods. This included aminoglycoside resistance genes (*aadA, strA, aac*(3)IV, *aadB, aphA1* and *aphA2)*, β‐lactam resistance genes (*bla*_TEM_ *, bla*_SHV_ *, bla*_OXA‐1_ *, bla*_CTX‐M_ group 1*, bla*_CTX‐M_ group 2*, bla*_CTX‐M_ group 9*, bla*_CMY_ *, bla*_FOX_ *, bla*_DHA_ *, bla*_NDM_ *, bla*_KPC_ *, bla*_OXA‐48_ *, bla*_IMP_ and *bla*_VIM_), tetracycline resistance genes (*tetA, tetB, tetC, tetW, tetO, tetK, tetL* and *tetM*), macrolides resistance genes (*erm*(A)*, erm*(B)*, erm*(C)*, erm*(F)*, erm*(T) and *erm*(X)), quinolone resistance genes (*qnrA, qnrS, qnrB* and *aac(6′)‐Ib‐cr)*, lipopeptides resistance genes (*mcr‐1* to *mcr‐8)*, sulfonamides resistance genes (*sul1, sul2* and *sul3*) and phenicols (*Catl, floR* and *cmlA)*.

Phenotype–genotype concordance was evaluated at the antibiotic class level. The percentage of isolates phenotypically resistant according to CLSI breakpoints and the percentage carrying one or more resistance genes screened for that class were calculated. These paired values were tabulated and used descriptively to judge concordance (Table 2). MDR (≥ 3 antibiotic classes) was also compared across final pathotypes (EAEC, STEC/EHEC, EPEC, ETEC and non‐diarrhoeagenic).

**TABLE 2:** Distribution of pathogenic *E. coli* pathotypes and phylogenetic classification.

		Phylogenetic groups								
Category	No. of isolates (%)	A	B1	B2	C	D	E	F	Cryptic clade I	Unknown
STEC/EHEC	20 (7.14)	12	7	0	0	0	1	0	0	0
EAEC	78 (27.86)	43	20	4	4	1	2	1	0	3
EPEC	11 (3.93)	7	1	0	2	0	0	1	0	0
ETEC	6 (2.14)	6	0	0	0	0	0	0	0	0
Non‐virulent	165 (58.93)	115	25	2	6	4	2	6	1	4

### 2.5 Statistical Methods

Statistical analysis was conducted using SPSS software (version 20.0; SPSS Inc., Chicago, Illinois, USA). Contingency table *χ^2^* tests were performed to compare the occurrence of each phenotypic or genotypic feature among the *E.coli* isolates. The result was considered to be significant at *p* ≤ 0.05.

## 3 Results

### 3.1 Isolation and Identification of *E. coli* Strains

A total of 280 *E. coli* isolates were obtained and identified from faecal samples collected in Xizang, China, with a 100% isolation rate. Among them, 57 isolates were isolated in 2021, 126 strains were isolated in 2022 and 97 in 2023. The sampling encompassed diverse regions in Xizang, including Linzhi, Changdu and Shannan. Physiological and biochemical identification of 280 bacterial isolates confirmed all as typical *E. coli*, demonstrating negative Simmons' citrate and Voges–Proskauer tests but positive indole and methyl red reactions.

### 3.2 Detection of Virulence Determinants by PCR and Phylogenetic Group Determination

**FIGURE 1:**
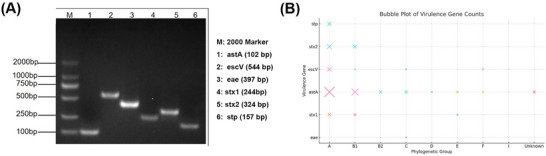
Molecular confirmation and phylogroup distribution of virulence genes in *E. coli* isolates from Tibetan pigs. (A) Multiplex PCR amplicons resolved on a 1.5% agarose gel. Lane M, 100–2000 bp marker; Lane 1, *astA* (102 bp); Lane 2, escV (544 bp); Lane 3, eae (397 bp); Lane 4, stx1 (244 bp); Lane 5, stx2 (324 bp); Lane 6, stp (157 bp). (B) Bubble plot depicting the distribution of six virulence genes across nine phylogenetic groups (A–F, cryptic clade I and unknown). Bubble area is proportional to the number of positive isolates (total *n* = 280); absence of a bubble indicates zero detections.

**FIGURE 2:**
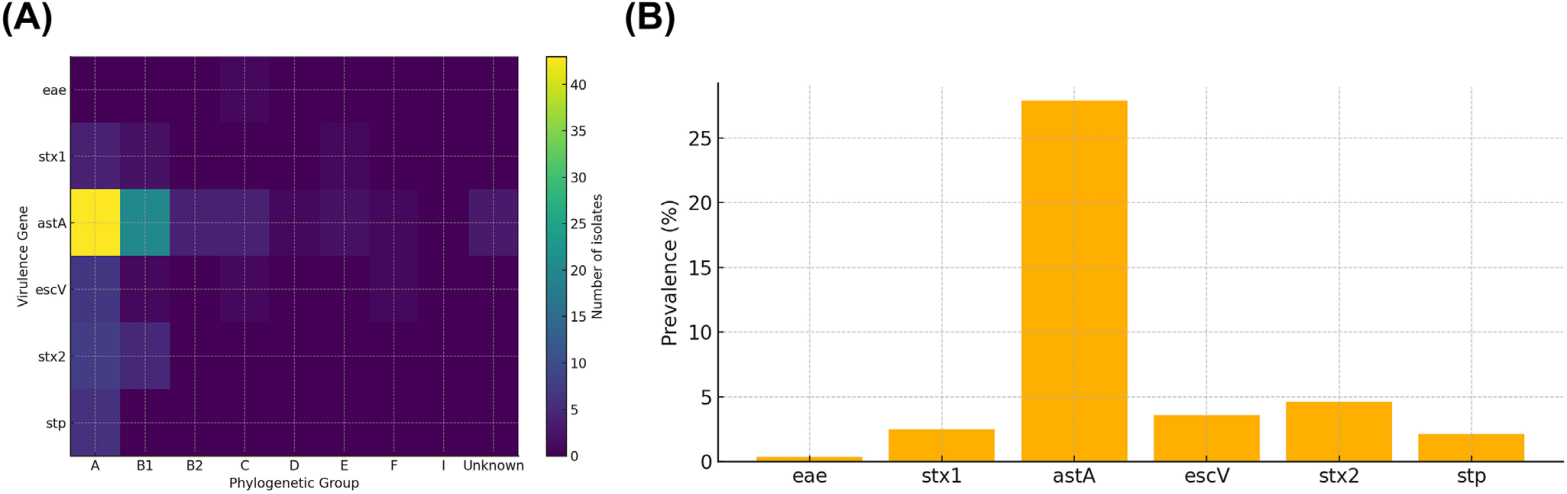
Virulence‐gene distribution in 280 *E. coli* isolates from Tibetan pigs. (A) Heat‐map of isolate counts for six virulence genes across nine phylogenetic groups (A–F, I and Unknown). (B) Overall prevalence of each gene expressed as a percentage of total isolates.

The 280 pathogenic *E. coli* isolates were tested for 13 virulence genes, with 6 different virulence genes being identified. Notably, *astA* exhibited a relatively high detection rate of 27.86%, whereas other virulence genes (*stx2, stx1, escV, stp* and *eae*) range from 0.36% to 4.64% (Figures 1 and 2). Conversely, virulence genes such as *bfpB, lt, sth, invE, ipaH, aggR* and *pic* were undetected. Further screening of these isolates for six specific virulence genes aimed to determine intestinal diarrhoeagenic *E. coli* pathotypes. The results revealed that 27.86% (78/280) of the isolates were classified as EAEC, 7.14% (20/280) as potential STEC/EHEC, 3.93% (11/280) as potential EPEC and 2.14% (6/280) as potential ETEC. Regarding phylogenetic grouping, most virulence genes belong to the A phylogroup, accounting for 65.36% (183/280) of the isolates. This was followed by B1—18.93% (53/280), B2—2.14% (6/280), C—4.29% (12/280), D—1.79% (5/280), E—1.79% (5/280) and F—2.86% (8/280); cryptic clade I and unknown phylotypes accounted for 2.86% (8/280) of the isolates (Table 2). Moreover, chi‐square tests showed that the *astA* gene was significantly over‐represented in phylogroup A compared with all other groups combined (*χ^2^* = 17.6, df = 1, *p* = 0.0003); no other virulence gene displayed a group‐specific bias (all *p* > 0.05).

### 3.3 Detection of Antibiotic Resistance Genes and Antimicrobial Susceptibility Testing

**FIGURE 3:**
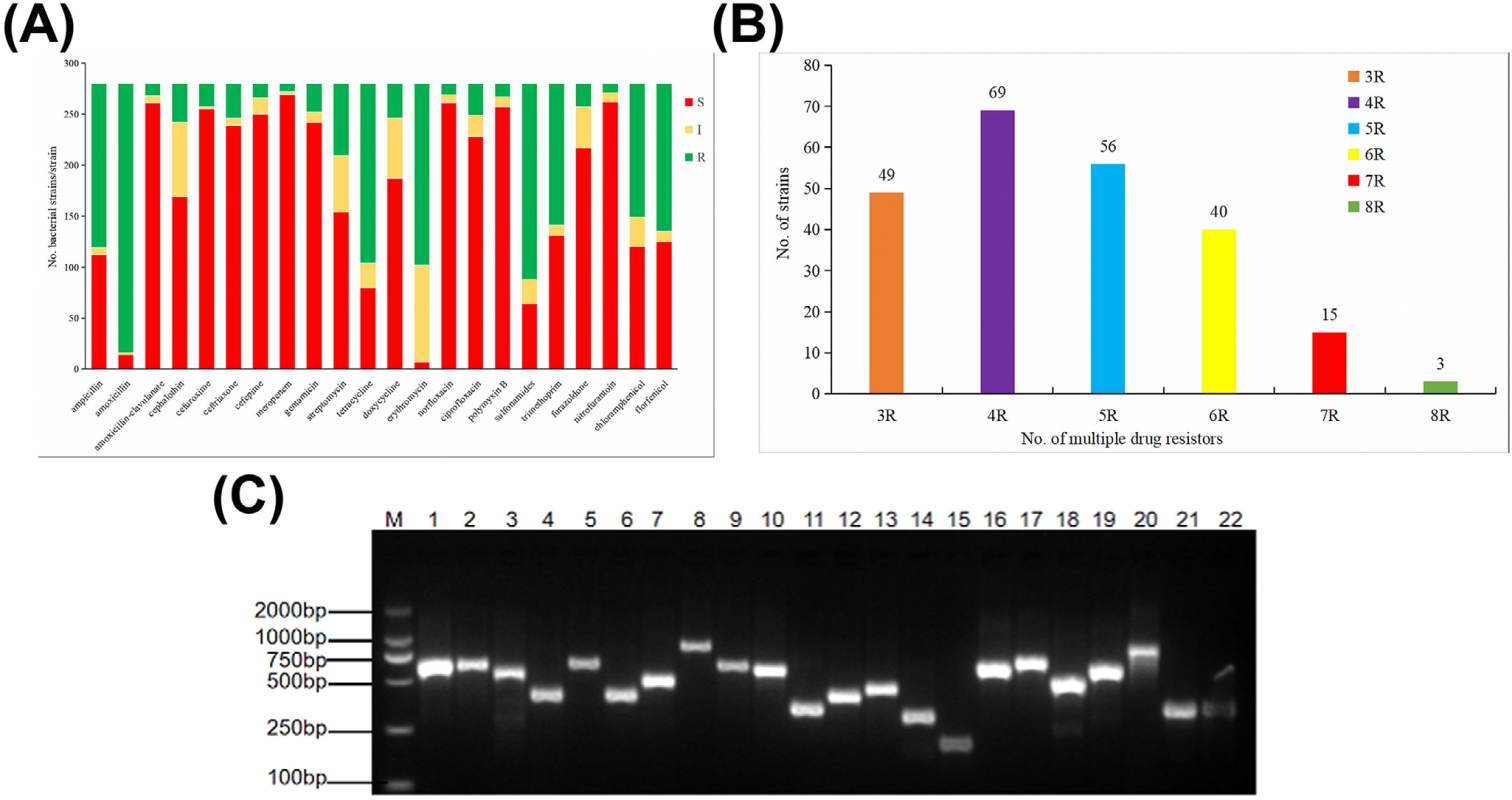
Antimicrobial susceptibility testing, multidrug resistance distribution and PCR detection of antibiotic resistance genes in *E. coli* isolates from Tibetan pigs. (A) Kirby–Bauer testing showed high resistance rates, especially to amoxicillin (94.29%) and sulfonamides (68.57%). (B) The multidrug resistance (MDR) illustrates *E. coli* distribution in various categories of resistances. (C) Detection of antibiotic resistance genes illustrates PCR profiles for the presence of resistance towards antibiotics in *E. coli* isolates. Lanes indicate individual resistance genes: bla_NDM_, bla_CTX‐M_ group 1, bla_CTX‐M_ group 9, *sul1*, *sul2*, *sul3*, *aadA*, *strA*, *aac*(3)IV, *aphA1*, *aphA2*, *qnrS, aac(6')‐Ib‐cr*, *mcr‐1*, *tetA*, *tetB*, *tetM*, *Cat1*, *floR*, *cmlA*, *erm*(B) and *erm*(T). 3R, 4R, 5R… = resistance to three, four, five and so forth antimicrobial classes, ≥ 8R = eight or more classes.

The antimicrobial resistance phenotype of the isolates to antibiotics are as follows: Ampicillin exhibited resistance in 57.50% (161/280) of isolates, amoxicillin in 94.29% (264/280), amoxicillin‐clavulanate in 4.29% (12/280), cephalothin in 13.57% (38/280), cefuroxime in 8.21% (23/280), ceftriaxone in 12.14% (34/280), cefepime in 5.00% (14/280), meropenem in 2.86% (8/280), gentamicin in 10.00% (28/280), streptomycin in 25.36% (71/280), tetracycline in 62.86% (176/280), doxycycline in 12.14% (34/280) (note: the “tetracycline” percentage refers only to tetracycline itself; doxycycline is reported separately and is not included in that class total), erythromycin in 63.57% (178/280), norfloxacin in 3.93% (11/280), ciprofloxacin in 11.07% (31/280), polymyxin B in 4.64% (13/280), sulfonamides in 68.57% (192/280), trimethoprim in 49.64% (139/280), furazolidone in 8.21% (23/280), nitrofurantoin in 3.21% (9/280), chloramphenicol in 46.79% (131/280) and florfenicol in 51.79% (145/280) (Figure 3A).

According to studies on antimicrobial agents, a significant proportion of 82.86% (232/280) of the *E. coil* isolates demonstrated MDR (Figure 3B). The distribution of MDR isolates was as follows: 3R in 17.50% (49/280), 4R in 24.64% (69/280), 5R in 20.00% (56/280), 6R in 14.29% (40/280), 7R in 5.36% (15/280) and 8R in 1.07% (3/280). Here, ‘XR’ denotes resistance to X distinct antimicrobial classes.

Among the various tetracycline resistance genes, *tetA* was the most prevalent, detected in 70.71% (198/280) of isolates, while *tetM* was the least prevalent at 16.79% (47/280). For the different phenicols resistance genes, *floR* was found in 57.14% (160/280) of the isolates, with *Catl* detected in only 1.79% (5/280). Among sulfonamide resistance genes, *Sul2* exhibited the highest detection rate of 37.50% (105/280), while *sul1* showed the lowest at 8.21% (23/280). The most common quinolone resistance gene was *qnrS*, detected in 45.71% (128/280) of isolates, with *aac (6’)‐Ib‐cr* being the least common at 5.36% (15/280). The aminoglycoside resistance gene *aadA* was the most prevalent, found in 35.71% (100/280) of isolates, while *aphA2* had a much lower prevalence of 2.50% (7/280). In terms of macrolides resistance genes, *erm*(B) was identified in only 7.14% (20/280) of isolates, and *erm*(T) in 4.64% (13/280). Among the different β‐lactam resistance genes, *bla*_CTX‐M_ group 9 exhibited the highest detection rate of 3.2% (9/280), while *bla*_CTX‐M_ group 1 showed the lowest at 0.36% (1/280). Additionally, *mcr‐1* was detected in 1.43% (4/280) of the various lipopeptides resistance genes identified (Tables 3 and 4) (Figure 3C). Hence, a staggering 94.29% of isolates were resistant to amoxicillin, underscoring widespread dissemination of antibiotic resistance in such a large population of Tibetan pigs. Besides, 82.86% of isolates exhibited MDR, and this fact necessitates immediate intervention in both local and global veterinary practice.

**TABLE 3:** Prevalence and phylogroup distribution of tetracycline and phenicol resistance genes in *E. coli* isolates from Tibetan pigs (*n* = 280).

			Phylogenetic group								
Antibiotic class	Gene	Isolates *n* (%)	A	B1	B2	C	D	E	F	I^a^	U
**Tetracyclines**	*tetA*	198 (70.71)	130	42	2	8	4	4	4	0	4
	*tetB*	123 (43.93)	84	16	4	8	2	2	3	1	3
	*tetM*	47 (16.79)	30	14	0	1	0	2	0	0	0
**Phenicols**	*floR*	160 (57.14)	115	26	1	9	2	2	3	0	2
	*cmlA*	88 (31.43)	61	18	2	4	0	1	0	0	2
	*catI*	5 (1.79)	4	0	0	1	0	0	0	0	0

^a^Cryptic clade I.

**TABLE 4:** Resistance genes for sulfonamides, quinolones, aminoglycosides, macrolides, lipopeptides and β‐lactams.

			Phylogenetic group								
Antibiotic class	Gene	Isolates *n* (%)	A	B1	B2	C	D	E	F	I^a^	U
**Sulfonamides**	*sul1*	23 (8.21)	17	3	0	2	1	0	0	0	0
	*sul2*	105 (37.50)	71	15	5	7	2	1	1	1	2
	*sul3*	97 (34.64)	73	14	2	3	1	1	1	0	2
**Quinolones**	*qnrS*	128 (45.71)	84	29	0	6	2	2	2	1	2
	*aac(6′)‐Ib‐cr*	15 (5.36)	12	0	1	1	0	0	1	0	0
**Aminoglycosides**	*aadA*	100 (35.71)	71	15	1	9	2	2	0	0	0
	*strA*	74 (26.43)	45	15	1	7	1	1	1	0	3
	*aac(3)IV*	22 (7.86)	17	5	0	0	0	0	0	0	0
	*aphA1*	40 (14.29)	32	1	0	2	3	1	1	0	0
	*aphA2*	7 (2.50)	5	2	0	0	0	0	0	0	0
**Macrolides**	*erm(B)*	20 (7.14)	17	2	0	1	0	0	0	0	0
	*erm(T)*	13 (4.64)	12	1	0	0	0	0	0	0	0
**Lipopeptides**	*mcr‐1*	4 (1.43)	0	4	0	0	0	0	0	0	0
**β‐Lactams**	*blaCTX‐M‐1*	1 (0.36)	1	0	0	0	0	0	0	0	0
	*blaCTX‐M‐9*	9 (3.21)	2	7	0	0	0	0	0	0	0
	*blaNDM*	7 (2.50)	7	0	0	0	0	0	0	0	0

^a^Cryptic clade I.

## 4 Discussion

In the present study, the molecular pathotyping of *E. coli* isolates from Tibetan pigs revealed EAEC as the predominant pathotype, accounting for 27.86% of the isolates. Potential STEC/EHEC followed at 7.14%, with the remainder comprising less than 5% of the isolates. Furthermore, the 280 pathogenic *E. coli* isolates belonged predominantly to phylogenetic groups A and B1, with a minority falling to groups B2, C, D, E and F. Consistent with the *χ^2^* outputs reported above, neither phylogroup nor pathotype explained the high MDR burden, confirming that resistance traits are disseminated broadly among Tibetan‐pig *E. coli* lineages. The current survey encompassed all nine intensive Tibetan‐pig farms operating in Xizang and spanned three consecutive years (2021–2023), offering a representative yet logistically feasible snapshot of antimicrobial resistance within this niche production system. Nevertheless, because no human, water or wildlife isolates were analysed, the present data reveal only the potential for zoonotic or reverse‐zoonotic exchange. Documented pathways include farmer exposure to pigs harbouring ESBL or *mcr*‐positive *E. coli* (Dohmen et al. 2023), contamination of surface waters used to irrigate smallholder fields (Sassi et al. 2025) and shared reservoirs such as synanthropic rodents or flies (Al Noman et al. 2024). Future One Health studies that pair pig, human and environmental sampling are required to quantify these links. Even so, national‐scale analyses of 1871 porcine isolates have revealed pronounced interprovincial heterogeneity in MDR *E. coli* clusters (Peng et al. 2022). A 20‐year meta‐analysis likewise revealed that trends in quinolone resistance in swine‐derived *E. coli* emerge only over extended timelines (Long et al. 2025). Adopting rolling, multi‐year surveillance frameworks similar to those advocated for Zhejiang herds (Yang et al. 2025) and extending sampling to backyard and pastoral systems across the Qinghai Xizang Plateau would therefore strengthen risk mapping and guide region‐specific stewardship.

A recent One Health investigation indicates that MDR *E. coli* can move from pigs to humans through three main pathways. First, farmers in close contact with pigs carry higher rates of ESBL and *mcr*‐positive *E. coli* than non‐farmers, supporting direct occupational transfer (Sudatip et al. 2023). Second, studies have recovered genetically similar resistant strains from slaughter pigs, retail pork and hospital patients, implicating the food chain as a conduit (Phomsisavath et al. 2024). Third, resistant bacteria and plasmids persist in manure‐amended soils, wastewater and surface waters, creating environmental reservoirs that recycle resistance back into the food web (Touati et al. 2025). Therefore, it must be emphasized that most clinically significant antimicrobial resistance arises from drug selection within human medicine and from healthcare‐associated transmission. Livestock to human transfer generally requires undercooking, raw product consumption or lapses in abattoir hygiene, and current industry practice separates routine antimicrobial administration from the preharvest period to reduce residues and microbial load. These documented pathways reinforce the public health relevance of the present findings.

*E. coli* is a ubiquitous bacterium in the intestines of both humans and animals, capable of causing a spectrum of infections ranging from mild gastrointestinal disturbances to severe urinary tract infections and even bloodstream infections (Johnson 2005; Ron 2010). A pivotal determinant of its pathogenicity is its virulence factors, among which the *astA* gene is particularly significant. The *astA* gene encodes heat‐stable enterotoxin 1, which stimulates fluid secretion in the small intestine and thereby induces diarrhoea (Maluta et al. 2016). Notably, *astA* was the most frequently detected virulence‐associated gene (27.86%) in our isolates. Because the animals sampled were clinically healthy, this prevalence is interpreted not as evidence of pathogenicity in pigs but as an indication that Tibetan pigs may act as reservoirs of *astA* positive *E. coli* with zoonotic potential. This high prevalence underscores its potential contribution to *E. coli* pathogenicity and highlights its importance as a critical virulence determinant. Conversely, other significant virulence genes, such as *stx2, stx1* (encoding Shiga toxins), *escV* (involved in the Type III secretion system), *stp* (encoding the serine protease autotransporter toxin) and *eae* (associated with attaching‐effacing lesions), showed lower detection rates, indicating their less frequent occurrence than *astA*. These findings provide valuable insights into the distribution and prevalence of specific virulence factors among diverse *E. coli* strains. Further investigation into these genes could yield effective strategies for the prevention, identification and treatment of associated diseases worldwide.

Antimicrobial resistance in animal bacterial populations poses a significant challenge to veterinary public health (Rolff et al. 2024). *E. coli*, recognized as a reservoir of antibiotic‐resistance genes, efficiently acquires and transfers these determinants to other pathogenic microorganisms within the intestinal tract (Durão et al. 2018). The global emergence and distribution of antibiotic‐resistant strains of *E. coli* are of grave concern, primarily due to the inappropriate and excessive use of antibiotics. A longitudinal study (2009–2021) on Chongming Island examined the prevalence of antimicrobial‐resistant *E.coli* isolates in food animals (Lv et al. 2022). The results revealed that over 90% of the isolates exhibited MDR phenotypes and demonstrated high resistance to phenicols, tetracycline, sulfonamides, penicillin, aminoglycosides and fluoroquinolones. Another investigation conducted on *E. coli* isolates from swine in Guizhou between 2013 and 2018 revealed a substantial preponderance of MDR. Among the 47 strains analysed, 97.9% (46/47) showed resistance to multiple drugs. Notably, 57.4% of these strains exhibited resistance to more than eight antimicrobials, with some demonstrating resistance to up to 16 immune agents (Yu et al. 2021), which aligns with the previous study (Gu et al. 2023). Our study identified the highest β‐lactam resistance, while the most frequently detected antibiotic resistance gene was *tetA*, indicating variation between antibiotic resistance phenotypes and the presence of antibiotic resistance genes. The differences in antimicrobial resistance rates may be attributed to the increased use of antibiotics for therapeutic targets and as growth promoters in intensive farming acts.

Furthermore, our study discovered the highest rates of antimicrobial resistance for amoxicillin, followed by sulfonamides, erythromycin, tetracycline, ampicillin and florfenicol contrast with previous reports on free‐ranging Tibetan pigs (Li et al. 2014). The variation in resistance rates between animals living freely and those raised intensively may be ascribed to factors such as the juxtaposition of these animals to human populations, environmental circumstances and livestock denseness. With the increased use of high‐dose antimicrobials in livestock farms, the problem of MDR bacteria is becoming increasingly severe (Price et al. 2015). In the present study, 82.86% of the *E. coli* isolates were resistant to more than three antimicrobial agents. This high MDR frequency did not differ by pathotype; ≥ 80 % of EAEC, STEC/EHEC, EPEC, ETEC and non‐diarrhoeagenic isolates showed the MDR phenotype, underscoring that virulence and resistance traits coexist in the same genetic backgrounds. Moreover, 2–14 antimicrobial resistance genes (ARGs) were detected in various strains, with over half exhibiting more than eight resistance genes. This highlights the substantial proportion and abundance of ARGs in these strains, emphasizing the magnitude of the drug resistance issue. The detection of drug‐resistance genes was predominantly concentrated in groups A and B1, suggesting that these groups are particularly susceptible to developing antibiotic resistance. Tetracycline has been historically and extensively used for treating bacterial infections in pigs, which may have contributed to the prevalence of antibiotic‐resistant strains (Pan et al. 2011). In this study, three tetracycline resistance genes were identified, *tetA* (70.71%, 198/280) and *tetB* (43.93%, 123/280) being common and considered the primary cause of tetracycline resistance in the strains. Also, after adjustment for group size, *tetA* carriage did not differ significantly across phylogroups (*χ^2^* = 9.51, df = 7, *p* = 0.22). The widespread prophylactic use of tetracycline has resulted in high concentrations of this antibiotic on farms. In addition to detecting tetracycline resistance genes, this study also found high grades of resistance to phenicols, with the detection rates of *floR* resistance genes showing high accord with the corresponding resistance phenotypes. This highlights the extensive presence of antibiotic resistance in intensively farmed settings due to the wide prophylactic use of antibiotics. Concordance analysis (Table 2) confirms that efflux‐mediated tetracycline and phenicol resistance is well captured genotypically, whereas β‐lactam and macrolide resistance involve additional, unscreened genes that warrant further investigation.

Furthermore, there is a concerning tendency in the growing surge of Gram‐negative bacteria developing resistance to β‐lactam drugs (Meini et al. 2019). It is noteworthy, however, that despite β‐lactams resistance rates being more pronounced than those for other antimicrobial agents in this study, this does not diminish the overarching significance and impact of antimicrobial resistance on public health. Additionally, the widespread use of colistin in the pig industry heightens concerns regarding its potential role in fostering antibiotic immunity (Cheng et al. 2021). The findings of this study underscore the pressing need for effective strategies and interventions to address and mitigate antibiotic resistance in both agricultural and clinical settings. The rapid horizontal distribution of *mcr‐1* through plasmids has posed challenges related to the growing prevalence of colistin resistance, particularly in livestock intended for human consumption. This phenomenon poses a significant risk to public health, as it facilitates the transmission of antibiotic‐resistant bacteria from animals to humans through the food chain. In our study, the detection rate *mcr‐1* was 1.43% (4/280), marking a decline compared to previously reported rates in Guizhou. Nevertheless, this still indicated colistin resistance and emphasized the necessity for ongoing surveillance and monitoring. In summary, these findings not only validate global antimicrobial concerns but draw attention to a critical lacuna in rural farm communities controlling zoonoses. Antibiotic MDR in the population of Tibetan pigs is a canary in a cage, an early sign and an early warning that warrants an immediate intervention at both national and global scales, through heightened surveillance and wise use of antibiotics. There are some limitations; for example, this survey drew exclusively on the nine intensive Tibetan‐pig farms in Xizang, giving a representative snapshot of antimicrobial resistance within that production system but not allowing inference about patterns that may exist in other provinces or in backyard and pastoral herds. The sample size was calculated using a prevalence‐based equation that meets current regulatory guidance but is not a formal power analysis; future work should adopt power‐driven designs to refine precision. Finally, because the study was cross‐sectional and focused solely on animal isolates, it yields no direct evidence of transmission to humans; integrated, longitudinal sampling of pigs, their environments and exposed human populations will be required to confirm and quantify the zoonotic pathways identification.

## 5 Conclusion

This study revealed a high prevalence of multi‐antibiotic resistance, carrying virulence genes among *E. coli* isolates from Tibetan pigs. The antimicrobial resistance profile of these *E. coli* isolates indicated an exceptionally high resistance to amoxicillin, followed by sulfonamides, erythromycin, tetracycline, ampicillin and florfenicol. Notably, the *tetA* gene emerged as the most predominant gene associated with antibiotic resistance. Furthermore, the significant detecting rate of the *astA* gene suggests its potential role in the pathogenesis of *E. coli*. Hence, the alarming prevalence of antibiotic‐resistant *E. coli* strains in Tibetan pigs underscores the urgent need for targeted interventions in veterinary medicine. The findings indicate a need for coordinated animal–human–environment surveillance rather than implying a direct causal chain from pig faeces to human infection. The study lays the groundwork for future research into zoonotic transmission pathways and antibiotic stewardship, which are essential to preventing the spread of MDR pathogens.

## Author Contributions

**Runbo Luo**: conceptualization, funding acquisition, formal analysis, investigation, methodology, visualization, writing – original draft, writing – review and editing. **Lihong Zhang**: conceptualization, funding acquisition, formal analysis, investigation, methodology, visualization, writing – original draft, writing – review and editing. **Kexin Li**: sample acquisition, writing – review and editing. **Yanan Zhong**: sample acquisition, writing – review and editing. **Peng Shang**: sample acquisition, writing – review and editing. **Md. F. Kulyar**: sample acquisition, writing – review and editing. **Suolang Sizhu**: conceptualization, funding acquisition, sample acquisition, investigation, methodology, writing – review and editing. **Ruibing Cao**: conceptualization, funding acquisition, sample acquisition, investigation, methodology, writing – review and editing. All authors have read and approved the final manuscript.

## Funding

This research was funded by the National Key Research and Development Program of China (2022YFD1600900) and special fund from the central finance to support the development and reform of local colleges and universities (YJSXK2025‐21).

## Ethics Statement

The authors have nothing to report.

## Consent

The authors have nothing to report.

## Conflicts of Interest

The authors declare no conflicts of interest.

## Data Availability

The data will be available on request to the corresponding author.
